# Changes of retinal microvascular parameters in patients with type 2 diabetes mellitus with or without diabetic kidney disease

**DOI:** 10.3389/fendo.2026.1727260

**Published:** 2026-02-25

**Authors:** Ruirui Ma, Chunwen Zheng, Yuling Niu, Duanrong Cao, Yijun Hu, Ling Jin

**Affiliations:** 1 Department of Ophthalmology, The People’s Hospital of Baoan Shenzhen, Shenzhen, China; 2 Department of Ophthalmology, The Second Affiliated Hospital of Shenzhen University, Shenzhen, China; 3 Department of Ophthalmology and Visual Sciences, The Chinese University of Hong Kong, Hong Kong, Hong Kong SAR, China; 4 Guangdong Eye Institute, Department of Ophthalmology, Guangdong Provincial People’s Hospital (Guangdong Academy of Medical Sciences), Southern Medical University, Guangzhou, China

**Keywords:** diabetes kidney disease, diabetic retinopathy, microvascular complications, retinal vasculature, type 2 diabetes mellitus

## Abstract

**Purpose:**

To investigate the quantitative retinal microvascular parameters in patients with type 2 diabetes mellitus (T2DM) with or without diabetic kidney disease (DKD).

**Methods:**

This retrospective study included 117 T2DM patients with DKD (DKD group) and 179 T2DM patients without DKD (non-DKD group). Quantitative retinal microvascular parameters were extracted from fundus photographs by an artificial intelligence (AI) system, including the number of retinal lesions (hard exudates, cotton wool spots, hemorrhagic spots, and microaneurysms), mean branch angle, fractal dimension (FD), mean vessel diameter (MVD), mean arterial diameter (MAD), mean venular diameter (MVeD), arteriole-to-venule ratio (AVR), mean vessel tortuosity (MVT), mean arterial tortuosity (MAT), mean venular tortuosity (MVeT), vessel density (VD), VD within the 3-mm and 5-mm foveal avascular zones (FAZ), and cup-to-disc area ratio (CDR). Systemic parameters were also collected, including systolic blood pressure (SBP), diastolic blood pressure (DBP), fasting plasma glucose (FPG), duration of T2DM, glycated hemoglobin (HbA1c), triglyceride (TG), total cholesterol (CHOL), bicarbonate (HCO3), blood urea nitrogen (BUN), uric acid (UA), creatinine, cystatin C, and estimated glomerular filtration rate (eGFR). Group comparisons were performed using t-test or Mann-Whitney test. Univariable and multivariable logistic regression analyses were used to determine the parameters associated with DKD.

**Results:**

Multiple retinal parameters were significantly different between the two groups, including numbers of hard exudates, cotton wool spots, microaneurysms, hemorrhagic spots, FD, MVD, MVeD, VD, and VD within the 3-mm and 5-mm FAZ (all P < 0.05). Univariable logistic regression analysis demonstrated significant associations between DKD risk and the following variables: SBP, BUN, HCO3, eGFR, creatinine, UA, cystatin C, duration of T2DM, staging of diabetic retinopathy (DR), number of cotton wool spots, microaneurysms, FD, MVD, MVeD, VD, and VD within 3-mm and 5-mm FAZ (all P < 0.05). Multivariable logistic regression analysis found that age (OR = 0.971, 95% CI: 0.948–0.994, P = 0.013), SBP (OR = 1.015, 95% CI: 1.002–1.029, P = 0.027), duration of T2DM (OR = 1.056, 95% CI: 1.019-1.095, P = 0.003), staging of DR (OR = 1.287, 95% CI: 1.064-1.557, P = 0.009), and VD within 3-mm radius of FAZ (OR = 0.000, 95% CI: 0.000-0.000, P < 0.001), were independent risk factors of DKD.

**Conclusion:**

Quantitative retinal microvascular parameters derived from fundus photographs show potential for identifying T2DM patients at risk of DKD, supporting the future use of retinal parameters as a non-invasive tool for early detection of renal impairment in patients with T2DM.

## Introduction

Type 2 diabetes mellitus (T2DM) is a globally prevalent endocrinological disorder, with its elevated incidence and high disability rate posing an urgent public health challenge to human health and the global economy. Studies estimate that the global prevalence of T2DM will reach 783.2 million individuals by 2045 (1, 2). Among its serious microvascular complications, diabetic kidney disease (DKD) is a leading cause of end-stage renal disease worldwide (3). DKD develops in approximately 30% to 40% of diabetic patients and is well-known to be associated with a markedly increased risk of mortality (4).

Current conventional screening for DKD primarily relies on invasive and resource-intensive methods, such as renal function tests and kidney biopsies. Pathophysiological studies indicate that prolonged hyperglycemia, hypertension, and oxidative stress in diabetic patients collectively contribute to systemic microvascular changes, including vascular remodeling and vasodilation, leading to renal impairment and other microvascular complications (5, 6). Similar risk factors also contribute to the retinal vascular changes. It was shown that increased nighttime SBP is a critical factor affecting retinal arteriovenous ratio in T2DM patients (7). Diabetes was shown to modify the associations between retinal venular tortuosity and cardiometabolic risk factors (8). Given the shared microvascular pathology, a strong correlation has been established between DKD and diabetic retinopathy (DR) (9). Retinal microvascular alterations have been shown to correlate with renal dysfunction, suggesting that retinal imaging could serve as a non-invasive surrogate for evaluating the renal involvement in patients with diabetes (10–12). A previous study has shown that both duration of diabetes and urinary albumin levels are significantly correlated with retinal mean vessel density of whole superficial capillary plexus in diabetic patients (10). In another study, estimated glomerular filtration rate was correlated significantly with macular avascular zones area in patients with T2DM (12). Moreover, the presence and severity of DR have been recognized as significant risk factors of DKD, highlighting the potential of retinal microvascular assessment in risk stratification for DKD in diabetic patients (13). Advances in artificial intelligence (AI) have also enabled the quantitative analysis of retinal microvascular parameters from fundus photographs, providing a rapid, non-invasive, and cost-effective imaging modality for screening.

This study aims to investigate the correlation between quantitative retinal microvascular parameters extracted from fundus photographs and the presence of DKD in patients with T2DM. Our findings may offer theoretical support for early detection of renal microvascular damage using accessible retinal imaging in this population.

## Materials and methods

### Study population

A total of 117 T2DM patients with DKD (DKD group) and 179 T2DM patients without DKD (non-DKD group) were recruited retrospectively from the Department of Ophthalmology at People’s Hospital of Baoan, Shenzhen between January 2022 and December 2024. The study protocol was approved by the Institutional Review Board (IRB) of People’s Hospital of Baoan Shenzhen and strictly adhered to the principles of the Declaration of Helsinki. Written informed consent was waived by the IRB, given that the study was conducted retrospectively and only involved existing clinical images and data, without compromising the patients’ privacy.

Medical records of patients with T2DM referred to our department for routine consultation were reviewed consecutively for inclusion. The inclusion criteria were as follows:

DKD group: T2DM patients with DKD diagnosed according to the KDIGO 2022 Clinical Practice Guideline for Diabetes Management in Chronic Kidney Disease, including elevation of urinary albumin-to-creatinine ratio (UACR) ≥ 3 mg/mmol, or a progressive decrease in eGFR < 60 mL/min/1.73 m^2^, or both, persistent for greater than 3 months (14–16).

Non-DKD group: T2DM patients who met the American Diabetes Association diagnostic criteria (17), but had no clinical evidence of DKD such as albuminuria and abnormal renal biochemistry (UACR < 3 mg/mmol, and eGFR ≥ 60 ml/min per 1.73 m^2^) (14–16).

Exclusion criteria applied to all participants included: 1) diagnosis of type 1 diabetes or other specific types of diabetes; 2) renal impairment attributed to non-diabetic etiologies; 3) severe hypertensive retinopathy; 4) coexisting any other types of ocular diseases (e.g., severe cataract, high myopia, optic neuropathy, glaucoma, uveitis, or other retinal vascular disorders such as retinal vein/artery occlusion); 5) history of any types of ocular surgery (e.g., vitrectomy); 6) history of any types of immune-related diseases (e.g., systemic lupus erythematosus).

### Data collection

Demographic information and clinical data of the patients were collected, including gender, age, duration of hypertension (HTN), and duration of T2DM. All patients underwent comprehensive ophthalmic examinations, including slit-lamp examination, best-corrected visual acuity, and intraocular pressure (IOP) measurement.

For retinal microvascular analysis, macula-centered 50-degree fundus photographs were captured from both right and left eyes using a Topcon fundus camera (TRC-50DX, Topcon Corperation, Tokyo, Japan) by trained ophthalmic technicians. The image from the right eye of every patient was selected for analysis; if its image quality was suboptimal, the image of the left eye was used instead. The severity of DR was graded using a standard protocol (18). Quantitative retinal microvascular parameters were extracted using the EVision AI System (19), which is an AI-based digital image analysis software used to quantify vascular features. The assessed parameters included: number of hard exudates, number of cotton wool spots, number of hemorrhagic spots, number of microaneurysms, mean branch angle, fractal dimension (FD), mean vessel diameter (MVD), mean arterial diameter (MAD), mean venular diameter (MVeD), arteriole-to-venule ratio (AVR), mean vessel tortuosity (MVT), mean arterial tortuosity (MAT), mean venular tortuosity (MVeT), vessel density (VD), VD within the 3-mm and 5-mm foveal avascular zone (FAZ), and cup-to-disc area ratio (CDR).

**Figure 1:**
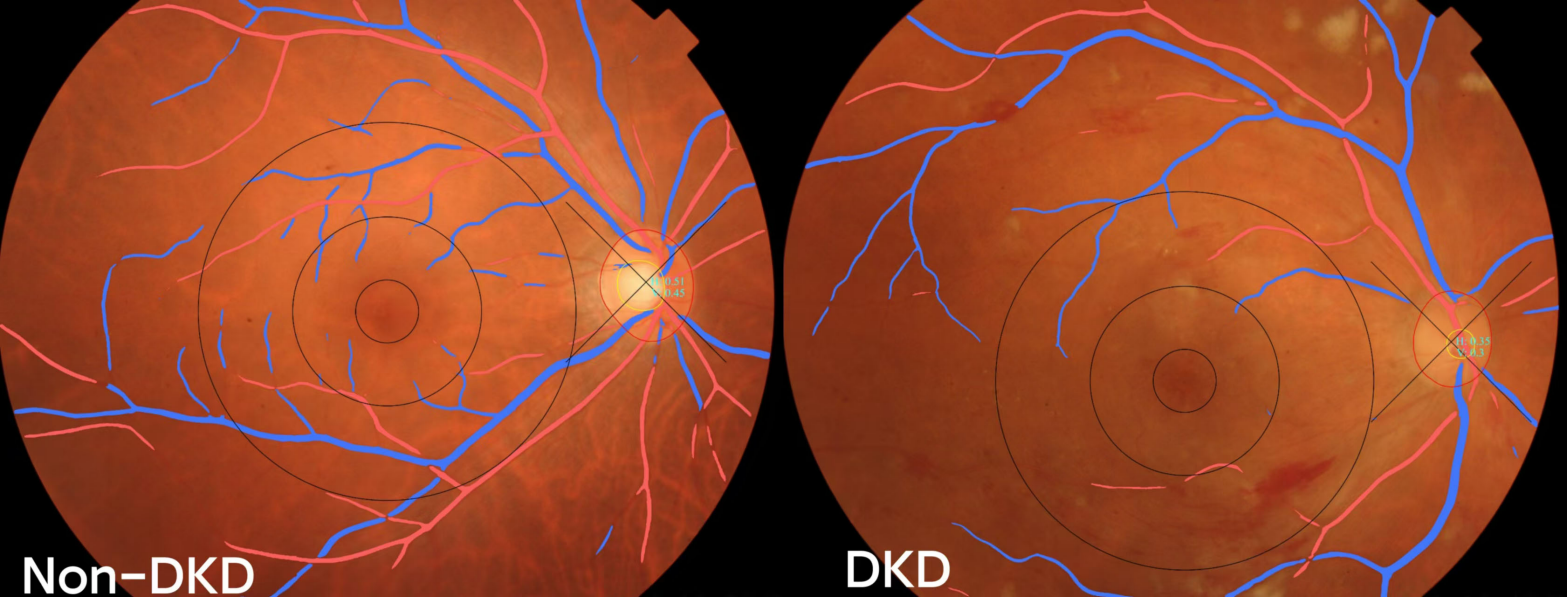
Segmentation of the retinal vessels in the two group.

The EVision AI System has been described in previous publications (19, 20). The fundus photos were processed by establishment the region of interest (ROI), denoising, normalization, and enhancement. Then the processed images were put into a deep learning network for segmentation of the retinal vessels. The quantitative retinal microvascular parameters were calculated and extracted as output. Segmentation of the retinal vessels in the two groups are shown in **Figure 1**.

Systemic parameters were measured from venous blood samples collected after an 8-hour fasting period. These parameters included systolic blood pressure (SBP), diastolic blood pressure (DBP), fasting plasma glucose (FPG), glycated hemoglobin (HbA1c), triglyceride (TG), total cholesterol (CHOL), bicarbonate (HCO3), blood urea nitrogen (BUN), uric acid (UA), estimated glomerular filtration rate (eGFR), and cystatin C.

### Statistical analysis

Group differences in demographics, medical history, systemic parameters, and retinal parameters were assessed. Categorical variables were presented as numbers and percentages and compared between groups using the Chi-square test. Continuous variables were tested for normality; those conforming to a normal distribution were expressed as mean ± standard deviation (SD) and compared using independent samples t-tests, while non-normally distributed data were presented as median and interquartile range (IQR) and compared using the Mann-Whitney U test.

To identify factors associated with DKD, univariable logistic regression analyses were first conducted. Variables showing statistical significance (P < 0.05) in the univariable analysis were subsequently included in a multivariable logistic regression model. The variance inflation factor (VIF) was calculated to assess multicollinearity among the included variables. A two-sided P value of less than 0.05 was considered statistically significant. All statistical analyses were performed using IBM SPSS Statistics version 24.

## Results

### Baseline characteristics

A total of 296 participants were included in this study, comprising 117 T2DM patients with DKD (DKD group) and 179 T2DM patients without DKD (non-DKD group). The comparative analysis of baseline demographic and systemic parameters is summarized in **Table 1**; **Supplementary Table S1**. Patients in the DKD group had a longer duration of T2DM and HTN, along with higher DR staging, SBP, TG levels, BUN levels, UA levels, and cystatin C levels, but lower HCO3- levels and eGFR (all P < 0.05). In contrast, no significant differences were found in age, gender distribution, DBP, CHOL, FPG, or HbA1c levels (all P > 0.05) between DKD and non-DKD groups.

**Table 1:** Descriptive analysis of baseline characteristics in the two groups.

Parameter	DKD group (n=117)	Non-DKD group (n=179)	P value
Gender (male/female)	81/36	115/64	0.377
SBP (mmHg)	136.00 (26.50)	128.00 (26.00)	**0.001**
DBP (mmHg)	79.56 ± 9.95	79.78 ± 10.41	0.856
Age (years)	56.63 ± 11.83	56.96 ± 11.48	0.813
TG (mmol/L)	1.80 (1.38)	1.55 (1.39)	**0.049**
CHOL (mmol/L)	4.85 (1.70)	4.88 (2.00)	0.849
BUN (mmol/L)	7.60 (4.95)	5.10 (2.04)	**<0.001**
HCO3 (mmol/L)	23.47 (4.29)	24.63 (3.49)	**0.002**
Creatinine (μmol/L)	108.10 (113.20)	68.65 (27.71)	**<0.001**
eGFR (ml/min/1.73 m^2^)	54.43 (58.15)	102.60 (33.11)	**<0.001**
UA (μmol/L)	401.10 (187.40)	357.90 (130.90)	**<0.001**
Cystatin C (mg/L	1.38 (1.30)	1.00 (0.24)	**<0.001**
FPG (mmol/L)	7.91 (5.56)	7.85 (4.98)	0.519
Duration of HTN (years)	0.00 (3.00)	0.00 (0.00)	**0.014**
Duration of T2DM (years)	10.00 (9.50)	6.00 (9.00)	**<0.001**
HbA1c (%)	8.30 (3.10)	8.50 (4.00)	0.301

Presented as mean ± standard deviation (SD) or median (interquartile range, IQR) and bold number indicates P < 0.05; DKD, diabetic kidney disease; SBP, systolic blood pressure; DBP, diastolic blood pressure; TG, triglyceride; CHOL, total cholesterol; BUN, blood urea nitrogen; HCO3, bicarbonate; eGFR, estimated glomerular filtration rate; UA, uric acid; FPG, fasting plasma glucose; HTN, hypertension; T2DM, type 2 diabetes mellitus; HbA1c, hemoglobin A1c.

### Comparisons of retinal parameters between DKD and non-DKD groups

The comparison of retinal vascular parameters between the DKD and non-DKD groups is detailed in **Table 2**. Significant differences were identified in several retinal parameters between the two groups. Specifically, the DKD group exhibited worse corrected visual acuity (LogMAR) and a greater number of retinal lesions, including hard exudates, cotton wool spots, microaneurysms, and hemorrhagic spots. Furthermore, retinal microvascular morphology and density also differed; the DKD group demonstrated a lower FD, a larger MVD and MVeD, lower overall VD, and lower VD within both the 3-mm and 5-mm radii of the FAZ (all P < 0.05). However, no statistically significant differences were observed between the two groups in IOP, mean branch angle, MVT, MAT, MVeT, AVR, or CDR (all P > 0.05).

**Table 2:** Comparative analysis of retinal parameters between the two groups.

Parameter	DKD group (n=117)	Non-DKD group (n=179)	P value
Corrected visual acuity	0.10 (0.22)	0.00 (0.10)	**0.005**
IOP (mmHg)	15.00 (4.75)	15.00 (4.00)	0.712
Number of hard exudates	2.00 (18.00)	0.00 (4.00)	**<0.001**
Number of cotton wool spots	0.00 (1.00)	0.00 (0.00)	**<0.001**
Number of microaneurysms	17.00 (23.50)	10.00 (16.00)	**<0.001**
Number of hemorrhagic spots	2.00 (11.00)	0.00 (3.00)	**<0.001**
mean branch angle	66.15 (7.44)	66.26 (7.26)	0.775
FD	1.51 (0.09)	1.53 (0.06)	**<0.001**
MVD (um)	62.54 (9.26)	60.51 (7.78)	**0.005**
MAD (um)	54.99 (9.23)	54.69 (7.79)	0.482
MVeD (um)	71.89 (11.44)	68.44 (10.05)	**0.001**
MVT	0.86 (0.16)	0.84 (0.08)	0.289
MAT	0.64 (0.11)	0.64 (0.15)	0.995
MVeT	1.01 (0.26)	0.98 (0.22)	0.536
VD	0.07 (0.03)	0.08 (0.02)	**<0.001**
VD within 3-mm radius of FAZ	0.05 (0.03)	0.06 (0.02)	**<0.001**
VD within 5-mm radius of FAZ	0.07 (0.03)	0.08 (0.02)	**<0.001**
AVR	0.76 (0.12)	0.78 (0.12)	0.102
CDR	0.22 (0.11)	0.22 (0.11)	0.192

Presented as mean ± standard deviation (SD) or median (interquartile range, IQR) and bold number indicates P < 0.05; DKD, diabetic kidney disease; IOP, intraocular pressure; FD, fractal dimension; MVD, mean vessel diameter; MAD, mean arterial diameter; MVeD, mean venular diameter; MVT, mean vessel tortuosity; MAT, mean arterial tortuosity; MVeT, mean venular tortuosity; VD, vessel density; FAZ, foveal avascular zone; AVR, arteriole-to-venule diameter ratio; CDR, cup-to-disc area ratio.

### Univariable logistic regression analysis of baseline characteristics between DKD and non-DKD groups

For systemic parameters, univariable logistic regression analysis revealed that several factors were significantly associated with the presence of DKD. As shown in **Table 3**, SBP (OR = 1.023, 95% CI: 1.009–1.037, P = 0.002), duration of T2DM (OR = 1.060, 95% CI: 1.022–1.099, P = 0.002), levels of serum creatinine (OR = 1.043, 95% CI: 1.030–1.056, P < 0.001), BUN (OR = 1.524, 95% CI: 1.337–1.737, P < 0.001), HCO3 (OR = 0.902, 95% CI: 0.839–0.971, P = 0.006), eGFR (OR = 0.954, 95% CI: 0.942–0.966, P < 0.001), UA (OR = 1.004, 95% CI: 1.002–1.006, P < 0.001).

**Table 3:** Univariable logistic regression analysis of baseline characteristics.

Independent variables	OR	95% CI	P value
Gender	1.252	(0.761, 2.059)	0.376
SBP (mmHg)	1.023	(1.009, 1.037)	**0.002**
DBP (mmHg)	0.998	(0.975, 1.021)	0.855
Age (years)	0.998	(0.978, 1.018)	0.812
TG (mmol/L)	1.042	(0.943, 1.150)	0.421
CHOL (mmol/L)	1.012	(0.892, 1.148)	0.853
BUN (mmol/L)	1.524	(1.337, 1.737)	**<0.001**
HCO3 (mmol/L)	0.902	(0.839, 0.971)	**0.006**
eGFR (ml/min/1.73 m^2^)	0.954	(0.942, 0.966)	**<0.001**
Creatinine (μmol/L)	1.043	(1.030, 1.056)	**<0.001**
UA (μmol/L)	1.004	(1.002, 1.006)	**<0.001**
Cystatin C (mg/L)	11.677	(4.882, 27.930)	**<0.001**
FPG (mmol/L)	0.973	(0.921, 1.029)	0.341
Duration of T2DM (years)	1.060	(1.022, 1.099)	**0.002**
HbA1c (%)	0.939	(0.850, 1.036)	0.209
Duration of HTN (years)	1.037	(0.989, 1.086)	0.132

Presented as Odds Ratio (95% confidence interval) with the two groups as the dependent variable; Bold number indicates P<0.05; CI, confidence interval; SBP, systolic blood pressure; DBP, diastolic blood pressure; TG, triglyceride; CHOL, total cholesterol; BUN, blood urea nitrogen; HCO3, bicarbonate; eGFR, estimated glomerular filtration rate; UA, uric acid; FPG, fasting plasma glucose; T2DM, type 2 diabetes mellitus; HbA1c, hemoglobin A1c; HTN, hypertension.

In contrast, no significant associations were observed between the risk of DKD and the following systemic parameters, including gender, DBP, age, TG, CHOL, FPG, duration of HTN, or HbA1c (all P > 0.05).

### Univariable logistic regression analysis of retinal microvascular parameters between DKD and non-DKD groups

For retinal parameters, findings of univariable logistic regression analysis were demonstrated in **Table 4**. Results showed that more serious Staging of DR (OR = 1.573, 95% CI: 1.325–1.867, p < 0.001), a higher number of cotton wool spots (OR = 1.360, 95% CI: 1.127–1.642, P = 0.001), a higher number of microaneurysms (OR = 1.011, 95% CI: 1.001–1.022, P = 0.037), lower FD (OR = 0.000, 95% CI: 0.000–0.002, P < 0.001), larger MVD (OR = 1.052, 95% CI: 1.017–1.089, P = 0.003), larger MVeD (OR = 1.044, 95% CI: 1.015–1.074, P = 0.003), lower VD (OR = 0.000, 95% CI: 0.000–0.000, p < 0.001), lower VD within 3-mm radius of FAZ (OR = 0.000, 95% CI: 0.000–0.000, p< 0.001), and lower VD within 5-mm radius of FAZ (OR = 0.000, 95% CI: 0.000–0.000, P < 0.001) were significantly associated with an increased risk of DKD (all P < 0.05).

**Table 4:** Univariable logistic regression analysis of retinal microvascular parameters.

Independent variables	OR	95% CI	P value
Corrected Visual acuity	1.178	(0.458, 3.033)	0.734
IOP (mmHg)	1.012	(0.939, 1.091)	0.754
Staging of DR	1.573	(1.325, 1.867)	**<0.001**
Number of hard exudates	1.004	(0.999, 1.009)	0.140
Number of cotton wool spots	1.360	(1.127, 1.642)	**0.001**
Number of microaneurysms	1.011	(1.001, 1.022)	**0.037**
Number of hemorrhagic spots	1.013	(0.997, 1.030)	0.108
mean branch angle	0.987	(0.953, 1.023)	0.478
FD	0.000	(0.000, 0.002)	**<0.001**
MVD (um)	1.052	(1.017, 1.089)	**0.003**
MAD (um)	1.019	(0.986, 1.053)	**0.253**
MVeD (um)	1.044	(1.015, 1.074)	**0.003**
MVT	3.009	(0.468, 19.329)	0.246
MAT	1.160	(0.178, 7.540)	0.877
MVeT	1.647	(0.437, 6.202)	0.461
VD	0.000	(0.000, 0.000)	**<0.001**
VD within 3-mm radius of FAZ	0.000	(0.000, 0.000)	**<0.001**
VD within 5-mm radius of FAZ	0.000	(0.000, 0.000)	**<0.001**
AVR	0.088	(0.008, 1.019)	0.052
CDR	0.280	(0.016, 5.034)	0.388

Presented as Odds Ratio (95% confidence interval, 95% CI) with the two groups as the dependent variable; Bold number indicates P < 0.05; CI, confidence interval; IOP, intraocular pressure; DR, diabetic retinopathy; FD, fractal dimension; MVD, mean vessel diameter; MAD, mean arterial diameter; MVeD, mean venular diameter; MVT, mean vessel tortuosity; MAT, mean arterial tortuosity; MVeT, mean venular tortuosity; VD, vessel density; FAZ, foveal avascular zone; AVR, arteriole-to-venule diameter ratio; CDR, cup-to-disc area ratio.

In contrast, retinal parameters such as corrected visual acuity (LogMAR), IOP, number of hard exudates, number of hemorrhagic spots, mean branch angle, MAD, MVT, MAT, MVeT, AVR, and CDR were not significantly associated with the incidence of DKD (all P > 0.05).

### Multivariable logistic regression analysis for the determinants of DKD

Variables that were significant in the univariable analyses were included in a multivariable logistic regression model to identify independent determinants of DKD (**Table 5**). After adjustment, five factors remained independently associated with the risk of DKD: a younger age (OR = 0.971, 95% CI: 0.948–0.994, P = 0.013), a higher SBP (OR = 1.015, 95% CI: 1.002–1.029, P = 0.027), a longer duration of T2DM (OR = 1.056, 95% CI: 1.019-1.095, P = 0.003), a higher DR staging (OR = 1.287, 95% CI: 1.064-1.557, P = 0.009), and lower VD within 3-mm radius of FAZ (OR = 0.000, 95% CI: 0.000-0.000, P < 0.001). Notably, retinal microvascular parameters, including gender, number of cotton wool spots, MVD, and MVeD, which were significant in univariable analysis, lost their independent significance in the multivariable model (all P > 0.05).

**Table 5:** Multivariable logistic regression analysis for the determinants of DKD.

Independent variables	OR	95% CI	P value
Age (years)	0.971	(0.948, 0.994)	**0.013**
Gender	0.700	(0.439, 1.114)	0.132
SBP (mmHg)	1.015	(1.002, 1.029)	**0.027**
T2DM duration (years)	1.056	(1.019, 1.095)	**0.003**
Staging of DR	1.287	(1.064, 1.557)	**0.009**
Number of cotton wool spots	1.138	(0.924, 1.401)	0.224
MVD (um)	1.024	(0.959, 1.094)	0.479
VD within 3-mm radius of FAZ	0.000	(0.000, 0.000)	**<0.001**
MVeD (um)	0.968	(0.914, 1.026)	0.274

Presented as Odds Ratio (95% confidence interval, 95% CI) with the two groups as the dependent variable; Bold number indicates P<0.05; CI, confidence interval; DKD, diabetic kidney disease; SBP, systolic blood pressure; T2DM, type 2 diabetes mellitus; DR, diabetic retinopathy; MVD, mean vessel diameter; VD, vessel density; FAZ, foveal avascular zone; MVeD, mean venular diameter.

## Discussion

DKD is a common and severe complication of diabetes, affecting approximately 40% of patients with diabetes (21). The pathophysiology of DKD is complex, involving multiple processes such as metabolic dysregulation, chronic inflammation, and fibrosis, contributing to substantial morbidity and mortality. The growing burden of DKD underscores the urgent need for identifying accessible, non-invasive assessment methods to evaluate the risks of DKD.

Given that retinal microvasculature can be observed directly and non-invasively, and DR and DKD are both microvascular complications of diabetes that share common mechanisms in microvascular injury pathways, retinal microvascular changes may serve as a window to systemic microangiopathy, including renal involvement (22, 23). Both complications arise from hyperglycemia-induced microvascular endothelial dysfunction, oxidative stress, inflammatory activation, and crosstalk between endothelial cells and pericytes/podocytes (24, 25). Previous studies have demonstrated a strong correlation between retinal microvascular alterations and the presence of DKD, with some evidence suggesting that DR may even precede clinical DKD onset (11, 12, 26–29). In particular, retinal microcirculatory impairment has been reported to occur before the development of microalbuminuria (10). Therefore, this evidence highlights the potential of retinal microvascular parameters in monitoring renal microvascular damage of DKD.

In this study, we investigated the changes in quantitative retinal microvascular parameters in patients diagnosed as T2DM with or without DKD. Significant reductions in VD and FAZ density were observed in patients in the DKD group compared to those in the non-DKD group. Multivariable logistic regression analysis revealed that VD within the 3-mm FAZ was significantly associated with the risk of DKD. This finding aligns with previous reports of decreased VD in patients with DKD from multiple population-based studies (30–32). The observed progressive capillary loss may be attributed to the complex mechanisms underlying DKD and its complications. The renin-angiotensin-aldosterone system exerts potent vasoconstrictive effects, potentially contributing to hemodynamic disturbances in retinal microvasculature (33). Oxidative stress and inflammatory responses in DKD may also accelerate retinal vascular endothelial injury (34). Additionally, as previously reported, anemia secondary to reduced erythropoietin production, uremic toxin accumulation, and dialysis in DKD may further exacerbate capillary damage (35).

Early endothelial dysfunction is considered a key initiating factor in diabetic microvascular complications (36). Consistent with this, our study found that significantly larger MVeD and MVD were observed in the DKD group compared to the non-DKD group. Univariable logistic regression analysis also indicated that MVeD and MVD were significantly associated with the risk of DKD. Similar findings were reported in previous studies demonstrating that retinal venular dilation positively correlates with DKD risk in diabetic patients (37, 38). This may be attributed to endothelial injury-mediated upregulation of vascular endothelial growth factor, leading to increased retinal vascular permeability, elevated intravascular blood volume, and vascular dilation with enhanced elasticity (39). Hyperglycemia reduces erythrocyte deformability and enhances aggregation, while endothelial injury further activates platelet and coagulation pathways, promoting a hypercoagulable state that may contribute to retinal venular dilation (40). Notably, our study observed significant differences in venular but not arterial diameter between DKD and non-DKD groups, which are consistent with other population-based studies (39, 40).

Systemic parameters also played a significant role. Previous studies have confirmed that hypertension exacerbates renal and retinal pathology in diabetic models (41). The coexistence of hypertension and diabetes significantly increases the risk of diabetic microvascular complications, including DKD and DR (42). In our study, we found that higher SBP and longer duration of T2DM were significantly associated with the risk of DKD, in line with prior studies (43). A UK prospective clinical trial confirmed that intensive blood pressure and glycemic control effectively reduce the incidence of DR and DKD (44). However, the precise mechanisms by which hypertension and diabetes interact to exacerbate DKD and DR remain unclear.

Retinal lesions were also evaluated in our study. Hard exudates, resulting from extravasation of fluid, lipids, and lipoproteins into the retinal layers from microaneurysms and dilated capillaries, were common in patients with DKD (45). Significant differences in the urine ACR were observed between groups with and without macular hard exudates (46). The underlying mechanisms linking renal dysfunction and hard exudates remain unclear but may involve concurrent injury to renal and retinal vascular cells, leading to blood-retinal barrier disruption and subsequent hard exudate formation (46). Interestingly, a previous study has reported resolution of hard exudates in the macular area after hemodialysis, suggesting that the formation of macular hard exudates may be partially associated with concurrent renal dysfunction (47). Similarly, cotton wool spot, which is a collection of axoplasmic material in the nerve fiber layer, is widely believed to be caused by retinal ischemia and is common in patients with DKD (48). It exhibits no specific differences in number, size, or distribution between diabetic or hypertensive patients alone, but is more prevalent when both conditions coexist (49).

We admit that this study has several limitations. First, its single-center, retrospective design, and relatively small sample size limit the generalizability. The small study population also limits our ability to balance the risk factors associated with retinal damage between the two study groups. Although we have used multivariable regression to adjust for the effects of these risk factors, the possible effects of selection bias should not be neglected. Future research should involve multi-center data with a prospective design and a larger population. Second, the study population consisted solely of Chinese individuals, and future multi-ethnic cohorts are needed to validate these findings. Additionally, we did not stratify patients by DKD severity. Hence, prospective studies including different stages of DKD and healthy controls would help clarify the progression of retinal changes. What is more, the standard protocol of fundus photography in our department was macula-centered, so we could only use the macula-centered image for analysis. Future studies using ultra-wide field imaging may provide more details about the retinal vascular changes in DKD. Last but not least, fluorescein fundus angiography (FFA) was not routinely performed in many of the study participants to evaluate the extent of retinal ischemia, either due to lower staging of DR or poor kidney function. Optical coherence tomography angiography (OCTA) was also not available in our department for more accurate measurement of the retinal vessels during the time of the study. In future studies, OCTA can be applied in these patients.

In conclusion, retinal abnormalities, specifically the VD within 3-mm FAZ and the staging of DR, are independently associated with renal impairment in patients with T2DM, after adjustment for important etiological confounding factors such as age, SBP, and duration T2DM. These findings support the potential use of AI-assisted fundus photograph analysis as a non-invasive tool for detecting renal microvascular damage in patients with T2DM, facilitating the identification of patients at higher risk of DKD for early intervention and improved risk stratification.

## Data Availability

The raw data supporting the conclusions of this article will be made available by the authors, without undue reservation.
