# Supplementary material for: Changes of retinal microvascular parameters in patients with type 2 diabetes mellitus with or without diabetic kidney disease
Source: Front Endocrinol (Lausanne). 2026 Feb 25;17:1727260. doi: 10.3389/fendo.2026.1727260 (PMC12975603; doi:10.3389/fendo.2026.1727260)
Supplement: Supplementary file 1 [file Table1.docx]

**Table S1. Diabetic retinopathy staging in the two groups**

| DR Staging | DKD Group (n=117) | Non-DKD Group (n=179) |  |
| --- | --- | --- | --- |
| No apparent retinopathy | 24 (20.5%) | 82 (45.8%) |  |
| Mild NPDR | 17 (14.5%) | 35 (19.6%) |  |
| Moderate NPDR | 26 (22.2%) | 24 (13.4%) |  |
| Severe NPDR | 23 (19.7%) | 24 (13.4%) |  |
| PDR | 27 (23.1%) | 14 (7.8%) |  |

DR, diabetic retinopathy; DKD, diabetic kidney disease; NPDR, nonproliferative diabetic retinopathy; DR, diabetic retinopathy; PDR, proliferative diabetic retinopathy.
